# Associations of locomotor task performance with static and dynamic balance in pre-school children aged 3–6 years: an age-adjusted cross-sectional study

**DOI:** 10.3389/fpubh.2026.1900272

**Published:** 2026-08-19

**Authors:** Jingwen Yang, Zhihua Yin, Jiajun Jiang, Jingjing Pei

**Affiliations:** 1 College of Physical Education and Health, East China Normal University, Shanghai, China; 2 College of Physical Education, Jilin University, Changchun, Jilin, China

**Keywords:** age adjustment, children aged 3–6 years, dynamic balance, locomotor tasks, partial correlation, static balance

## Abstract

**Objective:**

To examine the cross-sectional associations between field-based locomotor task performance and static and dynamic balance in preschool children aged 3–6 years, and to assess how alternative approaches to accounting for age affect the estimated correlations.

**Methods:**

We analyzed pre-intervention baseline data from a motor intervention project involving 60 children (30 boys and 30 girls) aged 3.4–5.7 years at a kindergarten in Yancheng, Jiangsu Province, China. Locomotor tasks comprised the 10-m shuttle run, standing long jump, consecutive two-footed jumps, and lateral movement; balance was assessed using the eyes-closed single-leg stance and balance-beam walk. Pearson correlations and 95% confidence intervals (CIs) were calculated. Sensitivity analyses included Spearman rank correlations, leave-one-out analyses, and within-age-group analyses. Model A adjusted for exact age and sex, whereas Model B adjusted for age group and sex. The Benjamini-Hochberg procedure was applied to nine primary comparisons.

**Results:**

In unadjusted analyses, all nine pairs of measures were exceptionally strongly correlated (|r| = 0.967–0.998). In Model A, eight associations remained statistically significant after correction, with the exception of the association between balance-beam time and 10-m shuttle-run time. All nine associations were significant in Model B. After further adjustment for exact age and sex within each age group, several correlations weakened or reversed direction.

## 1 Introduction

Fundamental movement skills provide an essential foundation for children's participation in physical activity and subsequent sport-specific development (17). They are commonly classified as locomotor, object-control, and stability skills (1). The four tests used in this study-the 10-m shuttle run, standing long jump, consecutive two-footed jumps, and lateral movement-all involve bodily displacement, but performance may also depend on speed, explosive strength, agility, coordination, and task comprehension. We therefore refer to them collectively as “field-based locomotor task measures” and do not treat performance on any single test as equivalent to an overall level of locomotor skill established using a validated standardized battery. Balance is a central component of stability skills and involves maintaining or restoring postural stability under stationary or moving conditions (2).

Between 3 and 6 years of age, fundamental movement skills and postural control develop rapidly (3). The way children integrate visual, vestibular, and proprioceptive information changes with age, and performance on both locomotor and balance tests generally improves as children grow older (4). Studies of preschool children further suggest that correlations among different types of balance are usually weak and vary by age group and specific test (5, 6). Age may therefore influence not only absolute test scores but also the estimated relationships among different skill measures.

Previous studies of fundamental movement skills have often reported locomotor, object-control, and balance scores separately. When children of different ages are pooled, however, a correlation coefficient combines between-age-group differences with individual variation among children of similar ages. In children aged 3–6 years, comparing unadjusted correlations with estimates obtained under different approaches to age adjustment can help determine whether strong overall correlations primarily reflect a shared developmental trend across ages or a consistent correspondence among similarly aged children. This statistical issue has received relatively limited attention and provides the main rationale for the present study.

Using pre-intervention baseline data from an existing quasi-experimental project, this study aimed to: (1) describe the demographic and anthropometric characteristics of the 60 children and the distributions of the six field-test measures; (2) examine unadjusted correlations between four locomotor task measures and static and dynamic balance; (3) estimate partial correlations after adjustment for exact age and sex; (4) compare exact-age and age-group adjustment and conduct within-age-group sensitivity analyses; and (5) examine the relationship between eyes-closed single-leg stance and balance-beam performance. Because this was a cross-sectional analysis, all findings are interpreted as associations, without inference regarding developmental direction or causality.

## 2 Participants and methods

### 2.1 Study design, participants, and recruitment

This cross-sectional study used baseline data from an existing motor intervention project. Only individual test records collected before the intervention were analyzed; subsequent group allocation and intervention effects were not considered. Baseline assessments were completed in November 2024 at a kindergarten in Yancheng, Jiangsu Province, China, with all testing conducted between 09:00 and 11:00.

Participants were recruited through stratified random sampling, with the junior, middle, and senior kindergarten classes serving as strata. The original project enrolled 60 children, including 30 boys and 30 girls. Exact age ranged from 3.4 to 5.7 years, with 20 children in each of the 3–4, 4–5, and 5–6-year age groups. Eligibility criteria were preschool age; written informed consent from a guardian; generally good health and the ability to understand and safely complete the tests; no apparent motor impairment; no serious bone or joint injury during the previous six months that could affect participation; and no participation in specialized extracurricular balance or locomotor training. Children were excluded if they were unwell on the day of testing, could not complete all baseline assessments, or did not meet the eligibility criteria. Developmental disorders, neurological conditions, visual or vestibular problems, and musculoskeletal disorders were reported by guardians.

The study protocol was reviewed and approved by the Medical Ethics Committee of the Second Hospital of Jilin University (approval no. [2024] Postgraduate Review 403). Written informed consent was obtained from the guardian of each child. Children could voluntarily withdraw or stop testing at any stage of the study.

### 2.2 Measures, test procedures, and scoring

The six field tests documented in the original intervention project were used to assess static balance, dynamic balance, and locomotor task performance: eyes-closed single-leg stance, balance-beam walk, 10-m shuttle run, standing long jump, consecutive two-footed jumps, and lateral movement. The scoring rules for the six tests are summarized in Table 1.

**Table 1:** Six field tests and their scoring rules.

Measure	Unit	Scoring rule
Eyes-closed single-leg stance	s	Longer of two valid trials; longer is better
Balance-beam walk	s	Shorter of two valid trials; shorter is better
10-m shuttle run	s	Shorter of two valid trials; shorter is better
Standing long jump	cm	Longer of two valid trials; longer is better
Consecutive two-footed jumps	s	Shorter of two valid trials; shorter is better
Lateral movement	repetitions	Mean count from two 15-s trials; higher is better

All six tests were administered in the same indoor venue and in the following order: eyes-closed single-leg stance, balance-beam walk, 10-m shuttle run, standing long jump, consecutive two-footed jumps, and lateral movement. Before formal testing, trained assessors gave standardized instructions and demonstrations. Testing began after each child had become familiar with the task. Children wore clothing and shoes suitable for physical activity, and safety personnel were present throughout. A 30-s rest was provided between the two formal trials of each test. All assessments were administered by uniformly trained staff according to standardized procedures.

Eyes-closed single-leg stance: The child stood on the preferred leg while lifting the other foot naturally off the floor and holding both arms away from the trunk. Timing began after a blindfold was applied. The trial ended if the supporting foot moved, the raised foot touched the floor, or the child used an external object for support. Each trial was capped at 10 s, and an assessor remained behind and slightly to the side of the child for safety. Each child completed two valid trials separated by 30 s; the longer time (s) was recorded.

Balance-beam walk: A beam 3 m long, 10 cm wide, and 30 cm high was used, with a 20 × 20 × 30-cm platform attached at each end. The child began on the starting platform and walked in a straight line along the beam to the finishing platform. The time required to complete the task was recorded. Each child completed two valid trials, and the shorter time (s) was retained.

10-m shuttle run: The one-way distance was 10 m; each child completed one out-and-back run for a total distance of 20 m. The child stood behind the starting line with the feet naturally staggered; the front foot could not touch or cross the line, and a crouch start was not permitted. The starter used the standardized command “Ready-go” and started the stopwatch on “go.” A fixed, easily touched, non-displaceable marker was positioned at the center of the turn line. The child had to touch the marker clearly with either hand before turning and running back. Timing stopped when the child's chest crossed the vertical plane of the start/finish line. Completion time (s) was recorded, with a shorter time indicating better performance. Each child completed two valid trials separated by 30 s, and the shorter time was retained.

Standing long jump: The child stood with both feet behind the take-off line, took off from both feet simultaneously, and landed forward. Distance was measured from the front edge of the take-off line to the heel closest to the line at landing and recorded in centimeters; a longer distance indicated better performance. A trial was invalid if the child stepped backward after landing or touched the floor with a hand. Invalid trials were repeated after a rest. Each child completed two valid trials separated by 30 s, and the longer distance was retained.

Consecutive two-footed jumps: Ten soft blocks (10 cm long, 5 cm wide, and 5 cm high) were placed 0.5 m apart, with a take-off line 20 cm before the first block. The child stood behind the line with the feet together. On command, the child took off and landed with both feet simultaneously while jumping consecutively over all 10 blocks. Timing began at take-off and stopped when the child cleared the tenth block and landed on both feet. Completion time (s) was recorded, with a shorter time indicating better performance. A trial was invalid if the child stepped on, struck, or displaced a block; went around a block; deviated from the course; or used a single-foot take-off. Invalid trials were repeated after a rest. Each child completed two valid trials separated by 30 s, and the shorter time was retained.

Lateral movement: A low wooden bar measuring 60 cm long, 4 cm wide, and 2 cm high served as the central obstacle. The child performed continuous two-footed lateral jumps between the areas on either side of the bar. One repetition was counted each time both feet completely cleared the bar and landed on the opposite side. Valid repetitions completed in 15 s were recorded, with a higher count indicating better performance. Each child completed two formal trials separated by 30 s, and the mean count was used. Non-compliant jumps were not counted, but timing continued.

Note: Invalid attempts on the balance beam, 10-m shuttle run, standing long jump, and consecutive two-footed jumps were not counted as valid trials and were repeated after the child had rested. For lateral movement, a noncompliant jump was not counted, but timing continued.

### 2.3 Data verification and statistical analysis

We first checked sample size, duplicate IDs, missing values, variable names, units, scoring direction, and value ranges. To identify possible ordering or derivation patterns, the data were divided into 12 strata based on original project group, sex, and age group. Monotonicity in each of the six test scores with exact age was examined within each stratum, yielding 72 checks; none showed a pattern opposite to the expected direction of improvement. Separate regression models were fitted using exact age, sex, original project group, and age group as predictors. Coefficients of determination for the six test scores ranged from 0.995 to 0.999, indicating an unusually regular data structure. All descriptive statistics and correlations reported here were recalculated from the 60 individual baseline records; summary results in the original workbook that could not be reproduced consistently from the individual-level data were not used. The available project files did not fully retain each child's two trial-level scores, paper records collected in the field, dates of birth, exact test dates, or original timing-device records. Our verification therefore assessed only the internal structure and statistical consistency of the available baseline data and cannot substitute for independent verification against the original field records.

Continuous variables are presented as mean ± standard deviation and range. BMI was used only to describe the sample and was not included as a covariate in the partial-correlation analyses. Because the primary focus was the effect of alternative approaches to age adjustment on correlation estimates, the partial-correlation models adjusted only for age and sex. Pearson correlations were first used to evaluate linear relationships among the six field-test measures. We report correlation coefficients (r), two-sided *P* values, and 95% CIs derived using Fisher's z transformation. Spearman rank correlations and leave-one-out analyses were also conducted to evaluate robustness to distributional characteristics and individual observations.

Partial correlations were calculated using the residual method to assess the independent linear association between two motor-test measures after adjustment for covariates. Two models were specified to compare alternative approaches to age adjustment. Model A adjusted for exact age as a continuous variable and sex (k = 2), thereby controlling the age gradient as closely as the available data allowed. Model B adjusted for two dummy variables representing age group and for sex (k = 3), reflecting the age-band adjustment commonly used in preschool research. We tested eight associations between four locomotor task measures and two balance measures, plus one association between the static and dynamic balance measures. The Benjamini-Hochberg procedure was applied separately within Models A and B to control the false discovery rate (FDR) across the nine comparisons.

To further assess residual confounding by exact age within age groups, we repeated the nine partial correlations within each age group while additionally adjusting for exact age and sex, and applied the Benjamini-Hochberg correction separately within each group. All analyses were performed using custom scripts in Python 3.12; Pandas 2.2.3 and NumPy 2.3.5 were used for data handling and matrix operations, respectively. Two-sided P values for Pearson and Spearman correlations were approximated using the t distribution with df = n−2; partial correlations used df = n–k−2. For unadjusted Pearson correlations, 95% CIs were calculated using Fisher's z transformation with a standard error of 1/√(n−3); for partial correlations, the standard error was 1/√(n–k−3). A two-sided *P* < 0.05 denoted nominal statistical significance, and *q* < 0.05 after Benjamini-Hochberg correction was the primary significance criterion.

## 3 Results

### 3.1 Participant characteristics and test results

Complete baseline records were available for all 60 children, with no duplicate IDs or missing values. The sample comprised 30 boys and 30 girls and 20 children in each of the 3–4, 4–5, and 5–6 year age groups; exact age ranged from 3.4 to 5.7 years. The largest absolute univariate standardized value across the six measures was 1.52, and the largest squared Mahalanobis distance was 15.82, below the critical value of 22.46 for six degrees of freedom at p=0.001. No extreme observation required removal. However, the covariance matrix of the standardized measures had a condition number of 12,056, indicating exceptionally high collinearity. Participant characteristics and test results are presented in Table 2. These diagnostics suggest that the pronounced covariation among the six measures was not driven by a single extreme observation. Nevertheless, the unusual regularity warrants further review of the scoring, data-transformation, and data-entry processes and should not currently be interpreted as a genuine and stable relationship among different aspects of children's motor performance.

**Table 2:** Participant characteristics and descriptive statistics for the six field tests.

Index	Unit	*N*	Mean ±SD	Range
Age	years	60	4.53 ± 0.80	3.4–5.7
Height	cm	60	102.73 ± 5.51	96.0–112.0
Weight	kg	60	16.49 ± 1.99	14.0–20.0
BMI	kg/m^2^	60	15.57 ± 0.21	15.16–15.94
Eyes-closed single-leg stance	s	60	3.73 ± 1.11	2.28–5.28
Balance-beam walk	s	60	10.64 ± 1.61	8.78–13.02
10-m shuttle run	s	60	10.48 ± 0.68	9.52–11.45
Standing long jump	cm	60	65.06 ± 6.25	56.8–74.5
Consecutive two-footed jumps	s	60	7.99 ± 0.82	6.88–9.15
Lateral movement	repetitions	60	13.32 ± 2.52	9.5–17.0

The sample included 30 boys and 30 girls and 20 children in each of the 3–4, 4–5, and 5–6 year age groups. BMI was recalculated from individual height and weight records.

### 3.2 Associations between locomotor task measures and static balance

#### 3.2.1 Unadjusted correlations

Pearson correlation analysis showed exceptionally strong associations between each of the four locomotor task measures and eyes-closed single-leg stance time (Table 3).

**Table 3:** Correlations between locomotor task measures and eyes-closed single-leg stance time.

Locomotor task measure	Pearson r	95% CI	*P* value	Spearman ρ	*P* value
10-m shuttle run	−0.996	[−0.997, −0.993]	1.55 × 10^−61^	−0.983	1.48 × 10^−44^
Standing long jump	0.997	[0.996, 0.998]	5.59 × 10^−68^	0.986	8.73 × 10^−47^
Consecutive two-footed jumps	−0.998	[−0.999, −0.997]	4.30 × 10^−72^	−0.998	1.65 × 10^−74^
Lateral movement	0.992	[0.986, 0.995]	1.40 × 10^−53^	0.986	3.95 × 10^−47^

*N* = 60. Longer eyes-closed single-leg stance time indicates better static balance; shorter completion times indicate better performance on the 10-m shuttle run and consecutive two-footed jumps; longer standing long-jump distance and a higher lateral-movement count indicate better performance.

Pearson r values for eyes-closed single-leg stance with the 10-m shuttle run, standing long jump, consecutive two-footed jumps, and lateral movement were −0.996, 0.997, −0.998, and 0.992, respectively; the corresponding Spearman ρ values were −0.983, 0.986, −0.998, and 0.986. All two-sided *P* values were < 0.001.

Given the direction of scoring, children who maintained the eyes-closed single-leg stance for longer generally performed better on all four locomotor tasks. After each child was omitted in turn, the absolute Pearson correlations remained between 0.992 and 0.998, indicating that no single observation drove the findings. The scatterplot nevertheless showed clearly separated clusters for the three age groups, suggesting that the unadjusted correlations captured both between- and within-age-group variation.

**Figure 1:**
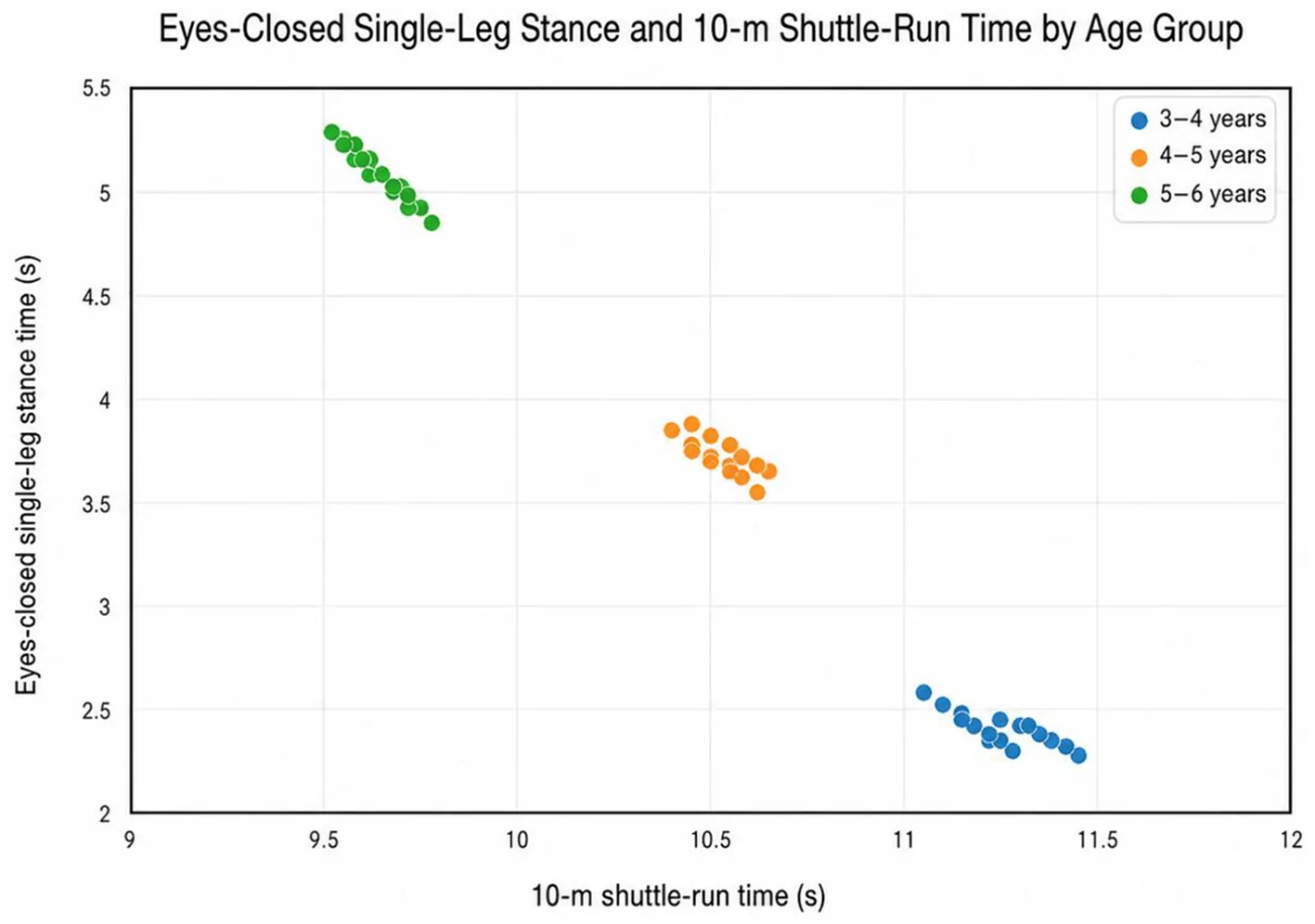
Scatterplot of eyes-closed single-leg stance time and 10-m shuttle-run time by age group (*N* = 20 per group).

To illustrate the overall distribution of the static-balance and locomotor measures, eyes-closed single-leg stance time was plotted against 10-m shuttle-run time (Figure 1).

Figure 1 shows that the three age groups had almost no overlap on either axis, although observations within each age group were also tightly arranged. An overall regression line across age groups was intentionally omitted to avoid misinterpreting between-group differences as relationships among children of the same age.

#### 3.2.2 Correlations after adjustment for age and sex

In Model A, partial r values for eyes-closed single-leg stance with the 10-m shuttle run, standing long jump, consecutive two-footed jumps, and lateral movement were −0.719, 0.850, −0.883, and 0.418, respectively; all adjusted q values were < 0.01. The corresponding partial r values in Model B were −0.784, 0.820, −0.959, and 0.793, with q < 0.001 for all. Although the direction of association was consistent across models, magnitude was sensitive to the way age was coded, with the largest between-model difference observed for lateral movement. The results from Models A and B are summarized in Table 4.

**Table 4:** Partial correlations between eyes-closed single-leg stance and locomotor task measures under alternative approaches to age adjustment.

Locomotor task measure	Model A partial r	95% CI	Model A q	Model B partial r	95% CI	Model B q
10-m shuttle run	−0.719	[−0.824, −0.566]	< 0.001	−0.784	[−0.867, −0.658]	< 0.001
Standing long jump	0.850	[0.758, 0.909]	< 0.001	0.820	[0.711, 0.890]	< 0.001
Consecutive two-footed jumps	−0.883	[−0.929, −0.809]	< 0.001	−0.959	[−0.976, −0.931]	< 0.001
Lateral movement	0.418	[0.180, 0.611]	0.001	0.793	[0.671, 0.873]	< 0.001

*N* = 60. Model A adjusted for exact age and sex; Model B adjusted for age group (categorical) and sex. The 95% CIs were calculated using Fisher's z transformation. Within each model, q values were adjusted across the nine primary comparisons using the Benjamini-Hochberg procedure.

### 3.3 Associations between locomotor task measures and dynamic balance

#### 3.3.1 Unadjusted correlations

Pearson correlation analysis showed exceptionally strong associations between all four locomotor task measures and balance-beam completion time (Table 5); Spearman correlations were in the same direction.

**Table 5:** Correlations between locomotor task measures and balance-beam completion time.

Locomotor task measure	Pearson r	95% CI	*P* value	Spearman ρ	*P* value
10-m shuttle run	0.967	[0.946,0.980]	2.92 × 10^−36^	0.994	7.02 × 10^−57^
Standing long jump	−0.970	[−0.982,−0.950]	3.04 × 10^−37^	−0.973	1.95 × 10^−38^
Consecutive two-footed jumps	0.988	[0.980, 0.993]	6.09 × 10^−49^	0.989	2.05 × 10^−50^
Lateral movement	−0.983	[−0.990, −0.971]	3.28 × 10^−44^	−0.993	2.23 × 10^−55^

*N* = 60. Shorter times indicate better performance on the balance beam, 10-m shuttle run, and consecutive two-footed jumps; longer standing long-jump distance and a higher lateral-movement count indicate better performance.

Pearson r values for balance-beam time with the 10-m shuttle run, standing long jump, consecutive two-footed jumps, and lateral movement were 0.967, −0.970, 0.988, and −0.983, respectively; the corresponding Spearman ρ values were 0.994, −0.973, 0.989, and −0.993. All two-sided *P* values were far below 0.001.

Given the direction of scoring, children who completed the balance beam more quickly generally performed better on all four locomotor tasks. In leave-one-out analyses, the absolute Pearson correlations remained between 0.966 and 0.989, providing no support for an explanation based on a single extreme observation.

**Figure 2:**
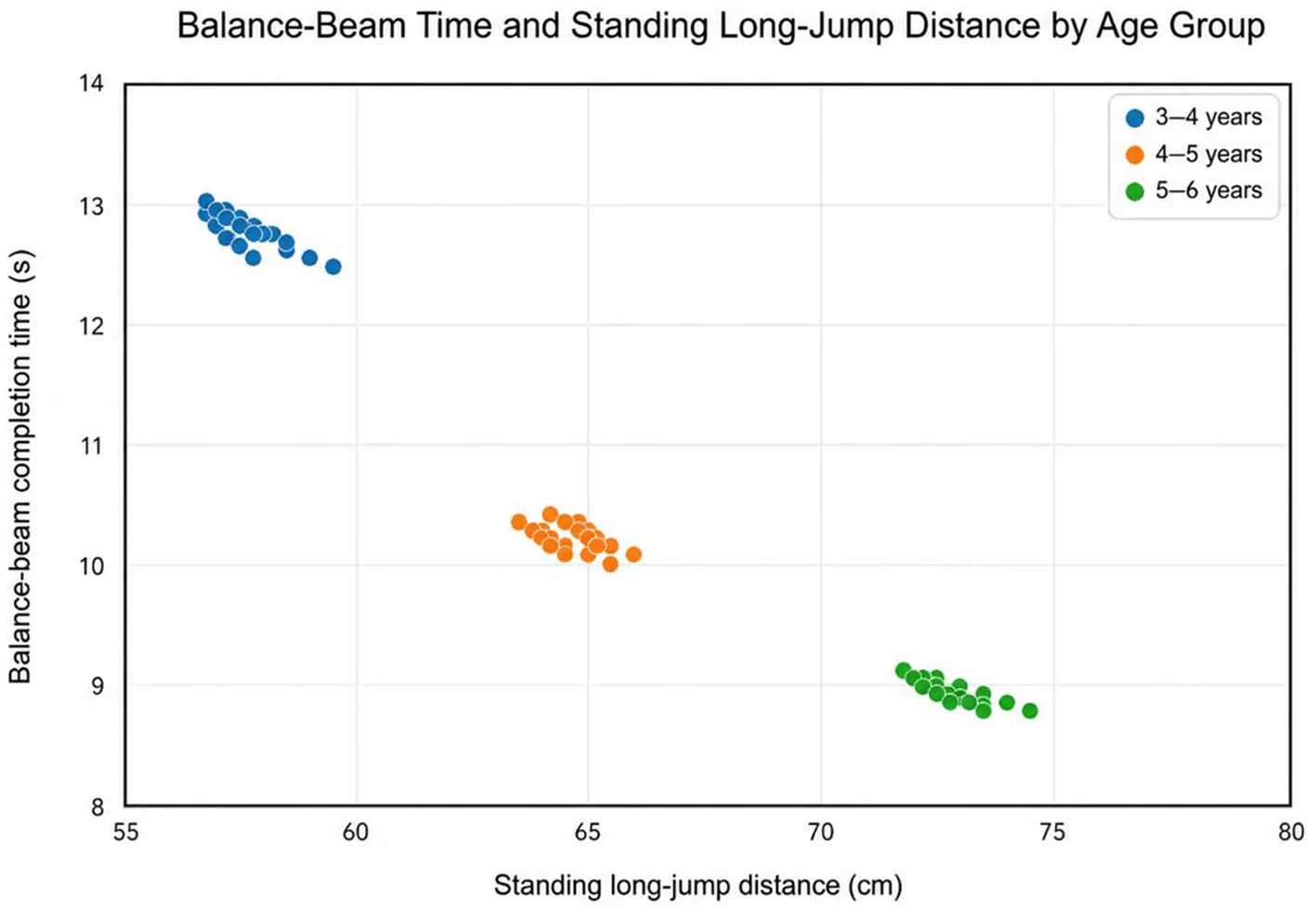
Scatterplot of balance-beam completion time and standing long-jump distance by age group (*N* = 20 per group).

To illustrate the overall distribution of dynamic-balance and locomotor measures, balance-beam completion time was plotted against standing long-jump distance (Figure 2).

Figure 2 shows that children with longer standing long jumps generally completed the balance beam more quickly in the full sample, but this overall pattern was accompanied by clear separation among age groups. No overall regression line is shown; Model A, which adjusts for exact age and sex, served as the primary analysis of the independent association.

#### 3.3.2 Correlations after adjustment for age and sex

In Model A, the partial correlation between balance-beam time and 10-m shuttle-run time was not significant (partial r = 0.209, q = 0.115). Balance-beam time was weakly negatively correlated with standing long-jump distance (partial r = −0.295, q = 0.028), strongly positively correlated with consecutive two-footed jump time (partial r = 0.865), and strongly negatively correlated with lateral-movement count (partial r = −0.825); q < 0.001 for the latter two associations. The results from Models A and B are summarized in Table 6.

**Table 6:** Partial correlations between balance-beam time and locomotor task measures under alternative approaches to age adjustment.

Locomotor task measure	Model A partial r	95% CI	Model A q	Model B partial r	95% CI	Model B q
10-m shuttle run	0.209	[−0.052, 0.444]	0.115	0.922	[0.871, 0.953]	< 0.001
Standing long jump	−0.295	[−0.514, −0.039]	0.028	−0.755	[−0.848, −0.615]	< 0.001
Consecutive two-footed jumps	0.865	[0.782, 0.918]	< 0.001	0.899	[0.834, 0.940]	< 0.001
Lateral movement	−0.825	[−0.893, −0.720]	< 0.001	−0.922	[−0.953, −0.871]	< 0.001

Note: N=60. Model A adjusted for exact age as a continuous variable and sex; Model B adjusted for age group (categorical) and sex. The 95% CIs were calculated using Fisher's z transformation. Within each model, q values were adjusted across the nine primary comparisons using the Benjamini-Hochberg procedure.

In Model B, partial r values for balance-beam time with the 10-m shuttle run, standing long jump, consecutive two-footed jumps, and lateral movement were 0.922, −0.755, 0.899, and −0.922, respectively, with *q* < 0.001 for all. The marked difference between Models A and B for the shuttle run indicates that adjusting only for broad age groups did not adequately remove the exact-age gradient within groups.

### 3.4 Association between static and dynamic balance

In the unadjusted analysis, eyes-closed single-leg stance time and balance-beam completion time were exceptionally strongly and negatively correlated (Pearson r = −0.979, *p* = 5.10 × 10^−42^; Spearman ρ = −0.987, p = 1.46 × 10^−47^). Given the direction of scoring, children who maintained the single-leg stance for longer generally completed the balance beam more quickly. These results are summarized in Table 7.

**Table 7:** Correlations between static- and dynamic-balance measures.

Analysis model	r or partial r	95% CI	*P or q*
Unadjusted Pearson correlation	−0.979	[−0.988,−0.966]	5.10 × 10^−42^
Unadjusted Spearman correlation	−0.987	—	1.46 × 10^−47^
Model A	−0.597	[−0.741,−0.400]	< 0.001
Model B	−0.818	[−0.889,−0.708]	< 0.001

*N* = 60. Model A adjusted for exact age and sex (k = 2); Model B adjusted for two age-group dummy variables and sex (k = 3). The 95% CIs were calculated using Fisher's z transformation with effective degrees of freedom adjusted for the number of covariates. Within each model, q values were adjusted across the nine primary comparisons using the Benjamini–Hochberg procedure.

The association remained negative in Model A (partial r = −0.597, 95% CI −0.741 to −0.400, q < 0.001) and was stronger in Model B (partial r = −0.818, 95% CI −0.889 to −0.708, q < 0.001). The difference between the coefficients suggests that the exact-age gradient within age groups may still affect the estimated strength of association.

**Figure 3:**
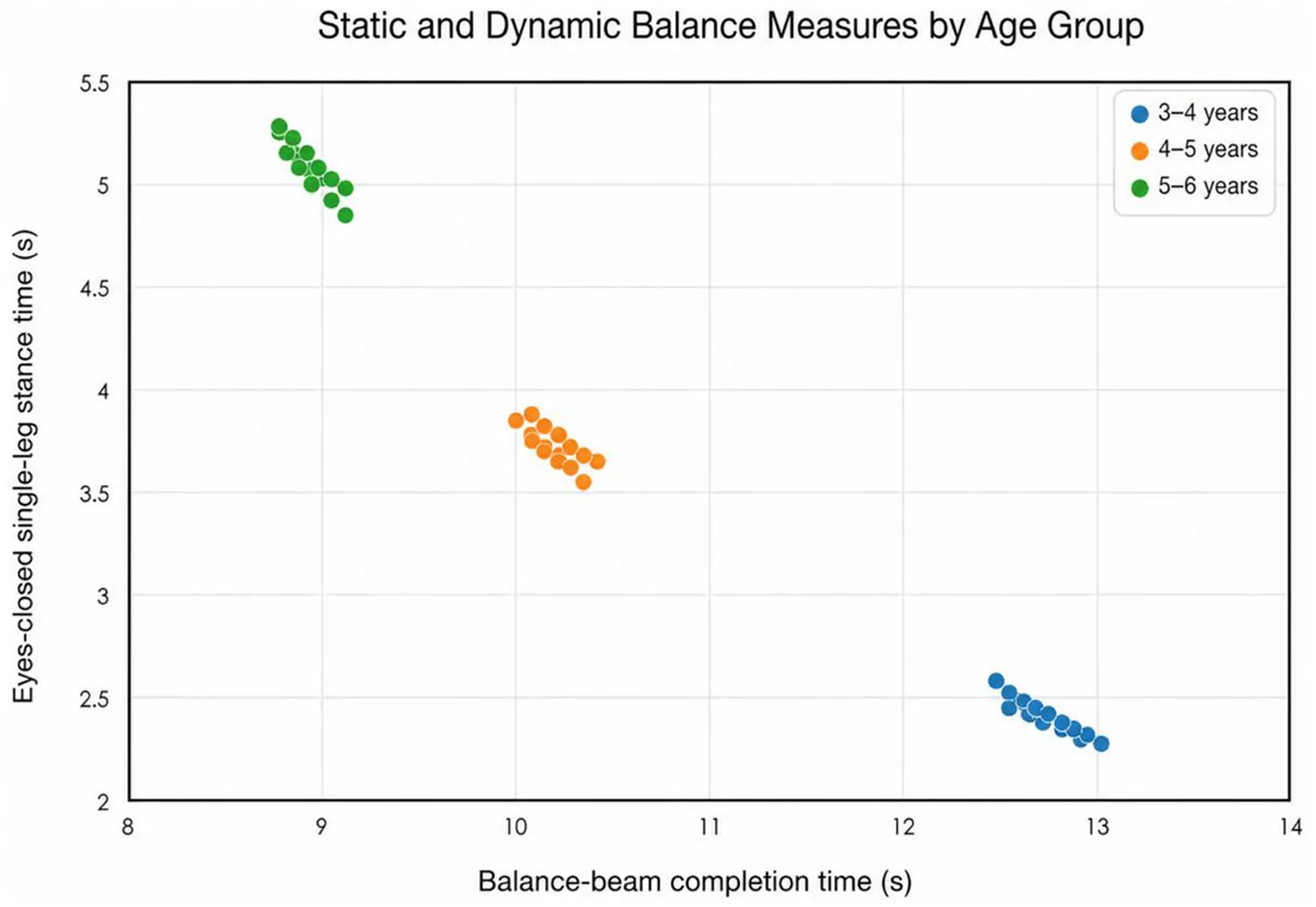
Scatterplot of eyes-closed single-leg stance time and balance-beam completion time by age group (*N* = 20 per group).

To show the overall distribution and age stratification of the two balance measures, eyes-closed single-leg stance time was plotted against balance-beam completion time (Figure 3).

Figure 3 shows clearly separated clusters for the three age groups, with observations also tightly arranged within each group. The overall negative correlation therefore reflects between-age-group differences and may also incorporate features related to exact age, scoring, or data processing within groups.

### 3.5 Within-age-group sensitivity analysis

After further adjustment for exact age and sex within each age group, the pattern of associations differed across groups (Table 8). All nine corrected associations were significant in the 3–4 year group. In the 4–5 year group, eight were significant; the association between eyes-closed single-leg stance and lateral movement was the exception, and several associations were opposite in direction to those in other age groups. In the 5–6 year group, only the associations between eyes-closed single-leg stance and the four locomotor task measures remained significant after correction. None of the associations between balance-beam time and the four locomotor measures, or between the two balance measures, was significant. These findings indicate that adjustment for broad age groups cannot replace adjustment for exact age; coefficients from small age-stratified samples should likewise not be interpreted as showing stable directions of association.

**Table 8:** Partial correlations within each age group after adjustment for exact age and sex.

Pair of measures	Age 3–4 partial r (q)	Age 4–5 partial r (q)	Age 5–6 partial r (q)
Single-leg stance−10-m shuttle run	−0.865 (< 0.001)	0.830 (< 0.001)	−0.924 (< 0.001)
Single-leg stance–standing long jump	0.916 (< 0.001)	0.955 (< 0.001)	−0.640 (0.010)
Single-leg stance–consecutive two-footed jumps	−0.957 (< 0.001)	−0.869 (< 0.001)	−0.962 (< 0.001)
Single-leg stance–lateral movement	0.901 (< 0.001)	−0.377 (0.123)	−0.913 (< 0.001)
Balance beam−10-m shuttle run	0.962 (< 0.001)	0.966 (< 0.001)	−0.293 (0.238)
Balance beam–standing long jump	−0.673 (0.002)	0.926 (< 0.001)	−0.299 (0.238)
Balance beam–consecutive two-footed jumps	0.948 (< 0.001)	−0.911 (< 0.001)	−0.338 (0.218)
Balance beam–lateral movement	−0.712 (0.001)	−0.662 (0.003)	−0.434 (0.108)
Single-leg stance–balance beam	−0.886 (< 0.001)	0.913 (< 0.001)	0.439 (0.108)

*N* = 20 per age group. Cells show partial r (q value) after adjustment for exact age and sex within that age group. Within each age group, q values were adjusted across nine comparisons using the Benjamini–Hochberg procedure.

## 4 Discussion

### 4.1 Influence of age adjustment on correlation estimates

The six field-test measures were strongly correlated in unadjusted analyses, but the magnitude of these correlations changed markedly under different approaches to age adjustment. After Model A adjusted for exact age and sex, most associations remained statistically significant, although several coefficients were substantially attenuated. Model B, which adjusted only for age group and sex, retained stronger correlations. After additional adjustment for exact age and sex within age groups, several associations weakened, became non-significant, or even reversed direction. Thus, the overall correlations appear to reflect not only statistical relationships among the motor tests but also the pronounced influence of the age gradient and the method used to account for age.

Several studies in young children support a systematic effect of age on test performance. In 150 children aged 3–5 years, De Oreo and Wade (7) found significant age differences in both dynamic and static balance. Li et al. (8) likewise reported age-related improvement across multiple static- and dynamic-balance tests in 619 Chinese children aged 3–6 years, with age effects exceeding most sex effects. In the present study, performance on several measures showed a clear age gradient. The strong correlations in the full sample therefore probably reflect a shared pattern of age-related improvement as well as any stable correspondence among different aspects of motor performance.

Age effects need not be simply linear. Iivonen et al. (9) reported that balance, running speed, and standing long-jump performance may follow different linear or non-linear trajectories in children aged 4–5 years, while Kokštejn et al. (10) found that age and sex differences in fundamental movement skills varied by task and age band. In this study, exact age spanned only 3.4–3.8, 4.3–4.7, and 5.3–5.7 years within the three respective groups, yet continuous age variation remained within each group. During this period of rapid development, treating exact age as a covariate is more effective in reducing residual age confounding than adjustment based only on yearly age bands. Model A was therefore treated as the primary analysis.

### 4.2 Locomotor task measures and static balance

The direction of the associations between the four locomotor measures and eyes-closed single-leg stance was consistent across Models A and B, but their magnitudes differed substantially, and the within-age-group analyses revealed heterogeneity after adjustment for exact age and sex. For example, single-leg stance and lateral movement were strongly positively correlated in the 3–4 year group, not significantly correlated in the 4–5 year group, and strongly negatively correlated in the 5–6 year group. Statistical relationships among motor tests may therefore depend on developmental stage, and the current findings do not establish stable, consistent correspondence across tests.

Differences in task demands may help explain the inconsistent adjusted results. The eyes-closed single-leg stance requires a child to maintain a one-legged posture on a fixed support surface without bodily displacement and with limited visual information. Consecutive two-footed jumps require repeated two-footed take-offs and landings while maintaining forward progression and rhythm over low obstacles. The shuttle run involves acceleration, deceleration, and turning; the standing long jump and lateral movement differ in movement direction, structure, and scoring. Performance may therefore be influenced by lower-limb strength, motor coordination, rhythm control, and task comprehension. Because these factors were not measured directly, associations among the test scores should not be interpreted as direct evidence of a shared neurophysiological mechanism.

Evidence in preschool children also underscores the need to distinguish developmental trends, measurement approaches, and specific tasks. Using process-oriented scoring, Foulkes et al. (11) assessed fundamental movement skills such as running, hopping, and galloping and found that mastery varied across tasks. Their hopping task was not equivalent to the timed consecutive two-footed jump used here; their findings demonstrate task specificity but do not provide a direct comparator for the present jumping test. Iivonen et al. (12) similarly found that associations with physical activity differed across locomotor skills. The near-perfect covariation observed here is substantially greater than would typically be expected from such task-specific evidence, reinforcing the need to review the scoring and data-generation processes.

### 4.3 Locomotor task measures and dynamic balance

Associations between the four locomotor measures and dynamic balance, assessed using the balance beam, were strongly influenced by the approach to age adjustment. The 10-m shuttle run provides the clearest example: both the unadjusted analysis and Model B (adjusted for age group and sex) indicated a strong positive association with balance-beam time, whereas the association was markedly attenuated and no longer statistically significant in Model A after adjustment for exact age and sex. Age-stratified analyses further showed that the association was mainly present among children aged 3–4 and 4–5 years and was not significant in the 5–6 year group. Thus, estimated relationships between locomotor performance and dynamic balance depend on how age is modeled. The strong correlations observed in the full sample may reflect shared rapid age-related development as well as relationships between different aspects of motor performance.

This pattern may reflect substantial differences in task demands. The balance-beam test requires continuous control of gait on a narrow support surface, ongoing stabilization of the center of mass, and fine postural adjustments. By contrast, the 10-m shuttle run requires rapid acceleration, deceleration, turning, and reacceleration; performance may depend not only on balance but also on speed, change-of-direction ability, motor coordination, and task comprehension. Although both are field tests involving locomotion, their motor-control demands are not identical. Once the shared developmental effect of exact age was further controlled, the common component of performance captured by the two tests was reduced, and the correlation weakened accordingly. Because speed, neuromuscular control, and sensory integration were not directly measured, this remains a task-based interpretation rather than evidence of mechanism.

Previous work also suggests that correlations among motor tests do not necessarily indicate a common underlying motor ability. Using the TGMD-3 and MABC-2 to assess locomotor skills, ball skills, and balance in children aged 5–6 years, Bai et al. (13) found improvement across multiple domains after a motor intervention. Chen et al. (14) reported bidirectional predictive relationships between fundamental movement skills and a composite measure of physical fitness, but did not assess static and dynamic balance separately. These studies support the possibility that different aspects of motor performance change together, but they do not establish that different field tests measure the same latent construct and cannot explain the near-perfect linear correlations in our unadjusted analyses.

Taken together, our findings suggest some statistical association between locomotor task performance and dynamic balance, but this association is age-dependent and jointly influenced by task characteristics and the method used to account for age. During the rapid motor development that occurs between 3 and 6 years, analyses based solely on overall correlations may overestimate relationships among different aspects of performance. Adjustment for exact age controls age confounding more fully than age-group adjustment and therefore provides a more informative estimate of independent relationships among motor tests. Larger longitudinal studies using standardized motor assessment tools are needed to examine the stability and reproducibility of associations between individual locomotor tasks and dynamic balance.

### 4.4 Static and dynamic balance

Eyes-closed single-leg stance time and balance-beam completion time were exceptionally strongly and negatively correlated in the full sample, and the association remained negative in Models A and B. After adjustment for exact age and sex within age groups, however, the association was strongly negative among children aged 3–4 years, strongly positive among those aged 4–5 years, and non-significant among those aged 5–6 years. Neither the overall nor full-sample partial correlations therefore establish a single stable relationship between static and dynamic balance across ages.

Early research showed that different balance tasks are not interchangeable in young children (7, 18). De Oreo and Wade used several balance-beam and balance-board tasks and found that performance varied by both age and task type (7). Using single-leg stance, tandem stance, balance-beam walking, and functional reach in the same sample of children aged 3–6 years, Li et al. (8) likewise found that age and sex effects varied by test. The inconsistent directions in our stratified analyses further support interpreting findings in relation to the specific task and age group.

More directly relevant correlational studies also support task specificity. Among 128 children aged 4–5 years, Liu et al. (5) found no significant associations between balance-beam completion time and several measures of static postural sway; most other correlations between static and dynamic measures were weak. In 619 children aged 3–6 years, Zhu et al. (6) similarly reported only small age-adjusted correlations among different types of balance, with patterns varying across age groups.

Eyes-closed single-leg stance and balance-beam walking differ in visual conditions, support state, form of movement, and scoring. The former requires maintaining a one-legged posture on a fixed surface, whereas the latter requires continuously changing the base of support on a narrow surface. Static and dynamic balance tests should therefore be reported separately in preschool assessments. Neither should be treated as a proxy for overall balance or combined into an unvalidated total score.

### 4.5 Measurement interpretation, data quality, and practical implications

Measurement instruments and data quality are critical boundaries for interpreting these findings. A review by Cools et al. documented substantial differences among preschool motor assessment instruments in measured constructs, task content, and process- vs. outcome-oriented scoring (15); agreement between MOT 4-6 and M-ABC was also limited (16). The applicability of MABC-2 has also been examined in preschool children (19). The time, distance, and count outcomes from the original project's field tests are not equivalent to TGMD-3 process scores or standardized scores from the MABC series. More importantly, every unadjusted |r| in this dataset exceeded 0.96 and the covariance-matrix condition number exceeded 12,000. Even in the absence of a single extreme observation, this degree of collinearity is well beyond what would usually be expected from evidence of task specificity.

At present, the practical implications are therefore limited to measurement and study design. Assessments of motor performance in children aged 3–6 years should use multiple tasks with clear operational definitions, report locomotor, static-balance, and dynamic-balance outcomes separately, and present both exact-age-adjusted and within-age-group results. Given the cross-sectional design and the sensitivity of several estimates to the method of age adjustment, these findings should not be used directly to prescribe kindergarten training programs.

The overall correlation coefficients observed here were markedly higher than those reported in most previous studies of motor ability in preschool children. Spearman correlations, leave-one-out analyses, multivariate outlier assessment, and alternative age-adjustment models did not identify a single observation that could account for this pattern, but correlations of this magnitude still require cautious interpretation. Their stability and reproducibility should be examined in larger samples from multiple regions using standardized motor assessments. Importantly, rank correlations, leave-one-out analyses, and outlier checks assess sensitivity to ordering, individual observations, and distributional assumptions; they do not verify the authenticity of the data. Without complete trial-level records, the possibility that ordering, transformation, variable labeling, or other data-management processes contributed to the unusual correlation structure cannot be fully excluded.

### 4.6 Limitations

This study has several limitations. First, the sample was drawn from a single kindergarten and included only 20 children in each of three age groups. Within-group adjusted models therefore contained relatively many parameters, and their coefficients may be unstable. Second, although exact age was recorded to one decimal place, dates of birth and testing were unavailable, precluding calculation of age in months. Third, although BMI was recalculated, the primary models did not adjust for BMI, physical activity, previous motor experience, attentional state, or task familiarity. Fourth, the six field tests were not administered as part of a complete TGMD-3, MABC-2, or MABC-3 battery, and the original project records did not fully document some procedures, equipment specifications, or invalid-trial criteria. Although Spearman, leave-one-out, and within-age-group analyses were performed, effects of scoring, transformation, or data entry cannot be excluded. Finally, the cross-sectional design cannot establish developmental sequence or causality. More broadly, the small single-site sample, unmeasured potential confounders-including body composition, habitual physical activity, prior motor experience, and test familiarity-and limitations in measurement and data processing constrain interpretation. BMI was not included in the adjusted models because the study focused specifically on how alternative treatments of age influenced correlation estimates; future studies could incorporate BMI and other anthropometric measures to further assess robustness. The findings should therefore be considered exploratory, and their reproducibility and generalizability require confirmation in larger independent samples. In addition, the available files did not fully retain trial-level scores, paper field records, participant linkage information, exact testing dates, or original timing-device records. It was therefore impossible to independently reconstruct how the two trials yielded each final score. This limitation does not prevent statistical description of the available baseline values, but it reduces the verifiability of the unusual correlation pattern.

## 5 Conclusions

Using pre-intervention data from 60 preschool children aged 3.4–5.7 years, this study found exceptionally strong correlations among field-based measures of locomotor task performance and static and dynamic balance in the full sample. Most associations remained statistically significant after adjustment for exact age and sex, but their magnitude differed markedly from estimates obtained by adjusting only for age group. After further adjustment for exact age and sex within age groups, several associations weakened, became non-significant, or reversed direction. Each field test should therefore be reported separately, and these findings should not be taken to indicate a common ability, shared mechanism, or causal training effect. Given the unusual regularity of the data and incomplete trial-level source records and data-processing documentation, the results should be regarded as exploratory. Their stability and reproducibility require confirmation in independent samples with clear data provenance and complete trial-level records.

## Data Availability

The original contributions presented in the study are included in the article/supplementary material, further inquiries can be directed to the corresponding authors.
